# Early life stress enhances the association between residential nature exposure and fasting blood glucose

**DOI:** 10.1371/journal.pone.0352771

**Published:** 2026-07-09

**Authors:** Aaron M. Eisen, Jose Guillermo Cedeño Laurent, John D. Spengler, George M. Slavich, Hector A. Olvera-Alvarez

**Affiliations:** 1 School of Nursing, Oregon Health & Science University, Portland, Oregon, United States of America; 2 School of Public Health, Rutgers University, Piscataway, New Jersey, United States of America; 3 T.H. Chan School of Public Health, Harvard University, Boston, Massachusetts, United States of America; 4 Department of Psychiatry and Biobehavioral Sciences, University of California, Los Angeles, California, United States of America; Methodist University Cape Fear Valley Health School of Medicine, UNITED STATES OF AMERICA

## Abstract

**Background:**

Emerging epidemiological evidence indicates that groups in low socioeconomic positions exhibit more pronounced health benefits from nature exposure compared to more privileged groups. We have previously posited one possible mechanism underlying this phenomenon through our framework: (susceptibility to stress) groups in low socioeconomic positions are often exposed to more early-life stressors, which can induce a lifelong susceptibility to stress through various neurobiological pathways; (environmental sensitivity) susceptibility to stress, traditionally understood as heightened reactivity to stressors, could also encompass enhanced responsivity to health-protective exposures, inducing greater risks in adverse environments, but also greater benefits in protective environments.

**Objective:**

Examine the moderation effect of early life stress on the association between residential nature exposure and fasting glucose.

**Methods:**

We assessed the impact of residential nature exposure (Normalized Difference Vegetation Index) on glucose dysregulation (elevated levels of fasting blood glucose) with a specific focus on the moderation effect of early life stress (Stress and Adversity Inventory for Adults) using baseline data from a cohort of 340 nursing students.

**Results:**

An initial analysis did not support our linear dose-response hypothesis. However, a theory-guided exploration revealed a significant curvilinear trend wherein participants with higher but also lower exposure to early-life stressors both exhibited lower levels of fasting glucose when living in greener neighborhoods. By contrast, for participants with relatively moderate early-life stressor exposure, there was no association between neighborhood greenness and fasting glucose.

**Discussion:**

Our findings contribute to growing evidence and further support the idea that increasing access to nature within disadvantaged neighborhoods could be an effective strategy to mitigate metabolic risks and attenuate health disparities among vulnerable populations. As the evidence for this framework expands, it could inform more targeted interventions that leverage individual differences in environmental sensitivity to promote health equity, ultimately providing more nuanced and socioeconomically attuned approaches to public health.

## Introduction

Emerging epidemiological evidence indicates that groups in low socioeconomic positions exhibit more pronounced health benefits from nature exposure (contact with natural green spaces) compared to more privileged groups [1–7]. This implies that increasing access to nature for disadvantaged communities could be a strategic approach to attenuate health disparities by alleviating the risks of chronic stressors among groups who are the most susceptible to stress [8–10]. However, the evidence supporting this possibility is mixed [11,12], including reports of null associations [13,14], and further research is thus needed to better understand the mechanisms underpinning this phenomenon [15]. Clarifying these mechanisms could reveal more effective pathways for reducing health disparities, as nature-based interventions offer a promising approach: they are passive, promoting health without requiring behavioral change; they are sustainable, often with low maintenance costs; and they can be implemented through public health policy to create lasting impacts.

### Theoretical framework

**Fig 1:**
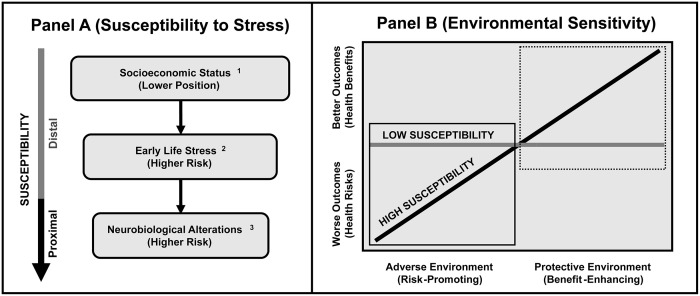
The integrative model of environmental sensitivity. Emerging epidemiological evidence indicates that groups in low socioeconomic positions exhibit more pronounced health benefits from nature exposure compared to more privileged groups (S1 Table). One possible mechanism underpinning this evidence is described in the Integrative Model of Environmental Sensitivity. Panel A: (Susceptibility to Stress) groups in lower socioeconomic positions often face higher exposure to early-life stressors, which can induce a lifelong susceptibility to stress through various neurobiological pathways. Panel B: (Environmental Sensitivity) individuals who are susceptible to stress are more responsive to stressors and health-protective exposures, inducing greater risks in adverse environments but also greater benefits in protective environments, relative to less susceptible individuals. (1) “Socioeconomic status” is defined as the position of an individual in their society, which is determined by social and economic factors that impact exposure to and experiences with psychosocial stressors. (2) “Early life stress” is defined as persistent exposure to psychosocial stressors during childhood, with ranging degrees of perceived severity, that induce neurobiological responses and could promote developmental alterations over time. (3) “Developmental alterations” are defined as enduring changes induced by early life stress across numerous neurobiological systems, which engage in multidirectional transactions throughout the lifespan, persistently amplifying crosstalk that increases reactivity to stressors. Within this framework, “susceptibility” refers specifically to heightened reactivity to stressors, while “sensitivity” expands this concept to consider enhanced responsivity to both stressors and health-protective exposures. This figure was adapted from “Susceptibility to Stress and Nature Exposure: Unveiling Differential Susceptibility to Physical Environments; a Randomized Controlled Trial” by Aaron M. Eisen, Gregory N. Bratman, and Hector A. Olvera-Alvarez, 2024, PLOS ONE, 19(4): e0301473. https://doi.org/10.1371/journal.pone.0301473.

One possible mechanism underpinning this phenomenon is described in the Integrative Model of Environmental Sensitivity (Fig 1 [15]). Central to the premise of this framework is the idea that groups in lower socioeconomic positions often face higher exposure to persistent psychosocial stressors in early life [16–23], which in turn could induce a lifelong susceptibility to stress through various neurobiological pathways (i.e., the Biological Embedding Model [24–33]). However, mounting evidence indicates that susceptibility to stress, traditionally understood as heightened reactivity to stressors, could also encompass enhanced responsivity to health-protective exposures, inducing greater risks in adverse environments, but also greater benefits in protective environments (i.e., the Differential Susceptibility Hypothesis [34–41]). Put together, this evidence implies that early life stress might be better understood as not merely increasing susceptibility to stress, but as cultivating a broader form of environmental sensitivity, enhancing responsivity to both stressors and protective environmental factors. This reframing provides a plausible mechanistic explanation as to why groups in lower versus higher socioeconomic positions could derive greater benefits from the health-protective effects of nature exposure.

In short, it is well-established that early life stress can induce developmental alterations that exert enduring effects, even decades later, across numerous neurobiological systems (e.g., central nervous system, autonomic nervous system, cardiovascular system, neuroimmune system, neuroendocrine system [42–51]). Together, these neurobiological systems engage in multidirectional transactions throughout the lifespan, persistently amplifying crosstalk that increases reactivity to stressors (e.g., Neuroimmune Network Hypothesis [52–54], Social Signal Transduction Theory of Depression [55–57]). For instance, mounting evidence indicates that early life stress sensitizes cortico-amygdala circuits with increased vigilance and threat processing, amplifying innate immune responses and facilitating the development of a chronic pro-inflammatory state (“brain to immune traffic” [52]). This amplified immune response also induces neuroinflammation that has been shown to further sensitize cortico-amygdala circuits with increased vigilance and threat processing (“immune to brain traffic” [52]) in a lifelong and self-perpetuating cycle. Neuroinflammation has also been shown to reduce cortico-basal ganglia reward sensitivity, eliciting profound changes in health behaviors and executive functioning [52]. These changes facilitate increased engagement with high-risk behaviors (e.g., tobacco, alcohol, and substance use, physical inactivity, poor-quality diet; Reward Deficiency Model of Addiction [58]) that in turn, further propagate inflammation.

Given that groups in lower versus higher socioeconomic positions often face higher lifetime stressor exposure (i.e., vulnerability) and are more sensitive to the health risks of chronic stress (i.e., susceptibility), early life stress could be a key pathway through which socioeconomic status becomes biologically embedded, altering how individuals experience and respond to environmental exposures across the lifespan and contributing to health disparities [59–66].

However, mounting evidence indicates that individuals who are susceptible to stress are also more responsive to protective environmental factors (e.g., Biological Sensitivity to Context Theory [67–69], Adaptive Calibration Model [70–72], Differential Susceptibility Theory [73–75]). Over the past two decades, the differential susceptibility paradigm has cultivated a very active area of research focused on disentangling person x environment interactions across three levels of analysis: genetic, physiological, and behavioral markers of differential susceptibility (for an excellent overview, see Boyce, 2016 [35]). As a culmination of this work, a seminal review of 56 studies encompassing thousands of participants (*n* ≈ 23,000) demonstrated that participants who were susceptible to stress across these biobehavioral markers also exhibited greater benefits in response to protective social factors (e.g., social support, positive feedback), relative to less susceptible participants [76]. Notably, these findings suggest that many of the susceptibility factors identified in the biological embedding literature may instead operate as differential susceptibility factors, including early life stress and a pro-inflammatory immune state.

In support of this argument, a national longitudinal survey of adults in the United States (*n* ≈ 34,000) found that participants who reported adverse childhood experiences exhibited greater decreases in transdiagnostic psychopathological factors following annual reductions in adult life stress, relative to participants who did not report adverse childhood experiences [77]. In terms of a pro-inflammatory immune state, one experimental study found that participants exposed to an in vivo inflammatory challenge (*n* = 61) demonstrated heightened neural activity in threat processing regions (bilateral amygdala and dorsal anterior cingulate cortex) when receiving negative versus neutral social feedback about their performance on a pre-recorded job interview, but also demonstrated heightened neural activity in reward processing regions (ventral striatum and ventromedial prefrontal cortex) when receiving positive versus neutral social feedback about the interview, relative to participants who were given a placebo (*n* = 57; [78]).

Put together, this evidence implies that groups with higher exposure to early-life stressors are more sensitive to both the health risks and benefits of their environmental conditions in a context-dependent manner. Therefore, it is plausible that groups in lower versus higher socioeconomic positions could derive greater benefits from the health-protective effects of nature exposure (e.g., Stress Reduction Theory [79,80], Attention Restoration Theory [81,82], Biophilia Hypothesis [83,84]) due to increased environmental sensitivity as a function of early life stress [15].

Notably, the evidence supporting this framework has centered on social environments, and further research is needed to determine whether increased environmental sensitivity induced by early life stress could also promote greater health benefits in protective physical environments such as nature. Eisen et al. used an experimental paradigm to evaluate this hypothesis and found that participants who were susceptible to stress (indicated by a pro-inflammatory immune state) exhibited greater autonomic recovery from an acute stressor in a virtual nature versus an office setting, relative to less susceptible participants [15]. However, even though a pro-inflammatory state is a well-established developmental alteration induced by early life stress [52–57,85–88], no differences in recovery were found for participants with higher versus lower exposure to early-life stressors.

The null findings for early life stress might have been attributed in part to the measure used (the Adverse Childhood Experience Questionnaire [89]) and limited variability in this sample of college-aged males (*n* = 64 [15]). For instance, this 10-item checklist might not have been comprehensive enough to detect differences in autonomic recovery, as it does not account for the subjective severity, frequency, timing, or duration of childhood stressors [90–92]. However, it is also possible that a pro-inflammatory state could be a more sensitive indicator of environmental sensitivity as a proximal measure, sharing a direct pathway between the indicator and outcome (see Fig 1). By contrast, self-reported assessments of early life stress are more distal measures, sharing an indirect pathway between the indicator and outcome that in turn, increases the risk of unmeasured confounders (see Fig 1). For instance, not all individuals with high exposure to early-life stressors develop susceptibility to stress due to protective factors and counteracting exposures such as parental attachment and social support [93,94], which could be immeasurable.

### Present study

Beyond the study by Eisen et al., to our knowledge, no other studies have examined whether early life stress may be associated with greater health benefits from nature exposure. Therefore, the purpose of the present study was to evaluate this association in a larger and more diverse sample (i.e., the Nurse Engagement and Wellness Study [95]), and using a more comprehensive measure of early life stress (i.e., the Stress and Adversity Inventory for Adults [96]).

In this context, we centered our investigation on glucose dysregulation, a risk factor for type 2 diabetes, which significantly contributes to the global burden of disease [97,98] and is more prevalent among groups in lower socioeconomic positions [99–102]. Evidence also indicates that residential nature exposure has protective effects on diabetes prevalence [103–106], incidence [107–110], insulin and glucose tolerance [111,112], and fasting blood glucose [111–115], with some studies showing stronger protective effects among groups in lower versus higher socioeconomic positions [3,5,116–118].

Building on this foundation, we used an observational paradigm to evaluate the effect of residential nature exposure (quantified using the Normalized Difference Vegetation Index across different radial buffers [250 m, 500 m, and 1000 m], centered on the residential address of each participant) on glucose dysregulation (elevated levels of fasting blood glucose), with a specific focus on the moderation effect of early life stress (before 18 years old). Based on the research summarized above, we hypothesized that adult participants with higher versus lower exposure to early-life stressors would exhibit stronger protective effects from residential nature exposure, as evidenced by lower levels of fasting blood glucose when living in greener neighborhoods.

Inherent to our hypothesis is the expectation that the association between early life stress and environmental sensitivity would be linear, increasing the protective effects of residential nature exposure in a dose-response pattern. However, we also conducted a theory-guided exploration to examine potential curvatures in the moderation effect of early life stress, as growing perspectives in the literature supporting our framework posit a curvilinear U-shaped association between early life stress and environmental sensitivity (the Biological Sensitivity to Context Theory [67–69] and the Adaptive Calibration Model [70–72]).

## Materials and methods

### Participants

Data were obtained from baseline measurements of a prospective cohort of men and women (*n* = 517; 18–55 years of age) enrolled during their first semester of the Bachelor of Science in Nursing program at the University of Texas at El Paso (i.e., the Nurse Engagement and Wellness Study [NEWS]; described in detail elsewhere [95]). Measurements included early life stress, residential nature exposure, fasting glucose levels, and relevant demographics. Participants were recruited using emails, posters, flyers, media outlets, and information sessions between May 17, 2016 and November 29, 2018. All participants provided written informed consent, and measurements occurred during an in-person visit to the Biobehavioral Research Laboratory. This study was approved by the institutional review board at the University of Texas at El Paso (857149-8).

### Study measures

#### Early life stress.

Early-life stressor exposure was assessed using the well-validated Stress and Adversity Inventory for Adults (STRAIN [96]). This dynamic and interview-based assessment tool quantifies exposure to different types of chronic (*n* = 29) and episodic (*n* = 26) stressors across various life domains (e.g., housing, education, employment, finances, relationships) and core social-psychological characteristics (e.g., interpersonal loss, physical danger, entrapment, humiliation) with known implications for health and wellness [119–121]. For each stressor endorsed, follow-up questions are used to ascertain the subjective severity, frequency, exposure timing, and duration of the stressor. This information is then used to calculate cumulative stress scores by summing the counts and perceived severity ratings of all stressors endorsed, including those in early life (before 18 years old) and adulthood (18+ years old). As research using this assessment tool has demonstrated that subjective stress severity is a more sensitive predictor of health outcomes (accounting for the “weight” of the stressors and capturing individual differences in susceptibility to stress [122]), early life stress was specified using the corresponding (time-specific) subjective severity index in our regression models.

#### Residential nature exposure.

Chronic exposure to residential nature (neighborhood-level green vegetation) was proxied using a cumulative opportunity approach across radial buffers centered on the residential address of each participant. Levels of green vegetation were quantified using the Normalized Difference Vegetation Index (NDVI; 30 m^2^ pixels [123]) from cloudless satellite images (Landsat 7 [124]) taken on a single summer day in 2016. Negative pixel values were present in a small portion of the region (< 5%) and were reclassified as missing data to avoid potential confounding effects of water bodies (e.g., rivers, irrigation canals). Pixel values were averaged across buffer sizes that corresponded to the amount of green vegetation within a 3–min (250 m), 6–min (500 m), and 12–min (1000 m) walk for a healthy adult in their mid-thirties [125]. This range of buffers was selected to focus on the local neighborhood, and based on systematic evidence that larger buffer sizes are less robust in predicting health [126]. Pixel values could range from zero (no green vegetation) to one (maximum green vegetation) and were specified as percentages in our regression models to reflect changes in fasting glucose levels relative to a 1% increase in residential nature exposure.

#### Fasting blood glucose.

Glucose measurements were obtained following an overnight fast from ~35 μL of whole blood (Lipid Panel + Glucose Panel Cassettes; range 2.8–27.8 mmol/L) using a Cholestech LDX Analyzer (Cholestech Corporation, Hayward, CA, USA). This instrument was calibrated before each measurement and used an enzymatic method (which has been shown to align with plasma concentrations using a hexokinase method; 95% CI: 92%–100% [127]). Glucose concentrations were expressed in mg/dL units, with higher values indicative of glucose dysregulation [128].

#### Demographic covariates.

Demographic covariates included years of age, biological sex, and self-reported income ratio (annual household income divided by the federal poverty threshold for household size). In addition, we included other relevant demographic covariates to eliminate the most plausible alternative explanations and confounders. First, as higher socioeconomic status during adulthood is unable to reverse or undo the lifelong effects of early life stress [129], we included the highest level of maternal education as an indicator of socioeconomic status during childhood (“How much education does/did your mother have?”: (1) “less than high school”, (2) “some high school”, (3) “high school graduate”, (4) “some college”, (5) “college graduate”). Second, as residential nature exposure could influence fasting glucose levels through promoting exercise and weight loss [130], we included the average duration of three common physical activities (walking, running, and biking) per week, during the past year (“During the past year, what was your average time per week spent at each of the following physical activities?”: (0) “0 min”, (1) “1–5 min”, (2) “6–20 min”, (3) “21–59 min”, (4) “1–2 hours”, (5) “3–6 hours”, (6) “7–10 hours”, (7) “11+ hours”; adapted from [131]) along with body mass index (kg/m^2^). Third, as our proxy for residential nature exposure was based on the current address of each participant, we included the duration of time that participants lived at their address (“How many years have you lived at your address?”).

### Analytical approach

We restricted the sample to participants under the age of 40, as with older age comes the development of aging-related health states and different behavioral patterns that could confound our analysis [132]. In this sample, the number of participants who were 40 years of age or older was relatively small (*n* = 19; 4%). We then excluded participants who were missing nature exposure data (*n* = 16; 3%), fasting glucose data (*n* = 28; 5%), and finally, several participants who did not complete the comprehensive early life stress assessment due to time constraints during the laboratory visit (*n* = 114; 22%). Therefore, the final analytical sample included 340 participants.

Our hypothesis was evaluated using linear regression models with continuous two-way interaction terms (Early Life Stress x Nature Exposure) to assess the moderation effect of early life stress on the association between residential nature exposure and fasting glucose. Interaction terms were tested across different buffer sizes for nature exposure (250 m, 500 m, and 1000 m) and were probed using Johnson-Neyman (J-N) intervals to determine the specific regions along the continuum of early life stress that significantly moderate the nature-glucose association. Models were adjusted for years of age, biological sex, household income ratio, maternal education, physical activity, body mass index, and years of residence.

Main effects were centered and variance inflation factor values provided no evidence of multicollinearity (cutoff value > 2.0). Assumptions for linear regression were affirmed and model fit was assessed prior to interpreting our results. We also tested the sensitivity of our results using models unadjusted for covariates and models with prominent outliers as determined by studentized residuals (cutoff value > |2.5|). Although we used alphas (0.05 level) to report significant results, our interpretations focused on effect sizes and confidence intervals across all models. Data were analyzed using R (4.4.1) with the “interactions*”* and “ggplot2*”* packages.

An initial analysis revealed no support for the linear dose-response hypothesis. Therefore, as a theory-guided exploration, we examined potential curvatures in the moderation effect of early life stress, as several of the core differential susceptibility theories supporting our framework posit a U-shaped association between early life stress and environmental sensitivity (the Biological Sensitivity to Context Theory [67–69] and the Adaptive Calibration Model [70–72]). Potential curvatures in the moderation effect were tested by including a quadratic term for early life stress. Likelihood ratio tests were used to determine whether including the quadratic term significantly improved model fit; otherwise, the model without the quadratic term was reported.

## Results

The sample consisted of 340 participants under the age of 40 (M = 24.0 ± 4.3 years of age; 77% female; Table 1). On average, participants lived at their reported address for over a decade (M = 10.4 ± 7.9 years). At the 250 m buffer size, the proportion of residential nature exposure ranged from 6% – 28% (M = 11% ± 3%), which was consistent across all buffer sizes. The results of bivariate correlation tests are provided in the supplemental materials (S1 Fig).

**Table 1:** Participant demographics.

Characteristic	M or (*N*)	SD or (%)	Observed Range
**Demographics**			
Years of Age	23.95	4.34	18.00–39.00
Female Bio-Sex	(262)	(77%)	---
Income Ratio^1^	2.30	1.87	0.06–12.44
Maternal Education^2^	3.63	1.27	1.00–5.00
**Physical Activity**			
Walking^3^	4.23	1.59	0.00–7.00
Running^3^	2.74	1.96	0.00–7.00
Biking^3^	1.00	1.78	0.00–7.00
**Health Indicators**			
Body Mass Index	25.34	5.34	14.10–51.90
Glucose (mg/dL)	90.93	10.07	69.00–142.00
**Early Life Stress**			
Stress Severity^4^	11.63	10.80	0.00–69.00
**Nature Exposure**			
NDVI (250 m)	10.78	3.21	6.38–27.77
NDVI (500 m)	10.83	2.89	6.77–25.17
NDVI (1,000 m)	10.90	2.61	6.78–25.85
Years of Residence^5^	10.39	7.87	0.00–28.00

^1^Self-reported annual household income divided by the federal poverty threshold for household size.

^2^Maternal educational attainment from, 1 “less than high school” to 5 “college graduate”.

^3^Average duration per week during the past year, from 0 “0 min” to 7 “11+ hours”.

^4^Stress and Adversity Inventory for Adults (STRAIN [96]).

^5^Duration of time that participants lived at their reported address.

NDVI, Normalized Difference Vegetation Index.

### Moderation effect of early life stress

We first tested the two-way interaction term (Early Life Stress x Nature Exposure) at the 250 m buffer size. The likelihood ratio test provided evidence that including a quadratic term for early life stress significantly improved model fit (*X*^*2*^ (2) = 11.66, *p* = .003). In the resulting model, we observed that the linear interaction was non-significant (*B* = 0.023, 95% CI [−0.009, 0.055], *p* = 0.162) whereas the quadratic interaction was significant (*B* = −0.003, 95% CI [−0.004, −0.001], *p* = .005), indicating a curvilinear trend in the moderation effect of early life stress.

**Fig 2:**
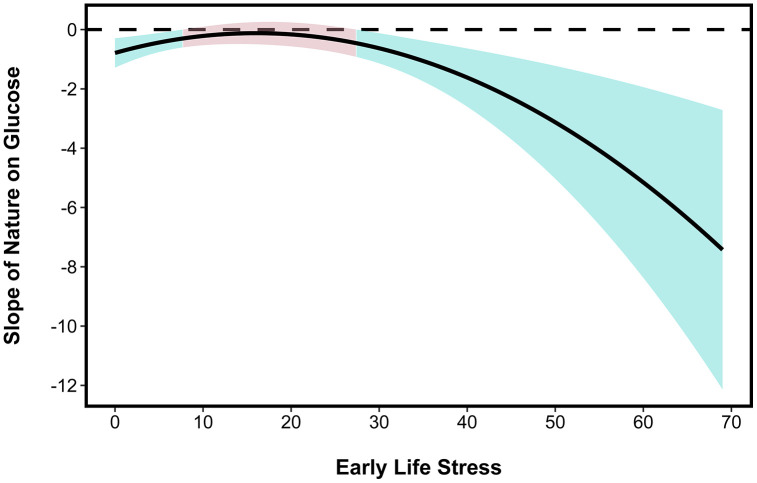
Moderation effect of early life stress on the nature-glucose association. Johnson-Neyman plot visualizing changes in the slope of nature exposure on fasting glucose levels (y-axis) along the continuum of early life stress (x-axis). Blue regions denote intervals where the moderation effect of early life stress is significant (*p* < .05). Estimates were obtained from a two-way interaction term (Early Life Stress x Nature Exposure).

To better understand the characteristics of this interaction effect, we plotted the slope of nature exposure on fasting glucose along the continuum of early life stress (Fig 2). In this plot, we observed that among participants with lower early-life stressor exposure, the protective effect of nature exposure on fasting glucose levels (negative regression slope) decreased (became less negative) as early life stress increased, an effect that was only significant (*p* < .05) when early life stress was within the interval of 0.0–7.7 points. We then identified a turning point (the vertex of the parabola) at 16.0 points (4.4 points above the sample mean), where the moderation effect changed direction. Specifically, we observed that among participants with higher early-life stressor exposure, the protective effect of nature exposure on fasting glucose levels (negative regression slope) increased (became more negative) as early life stress increased, an effect that was only significant (*p* < .05) when early life stress was within the interval of 27.5–69.0 points.

To understand the magnitude of these interaction effects, we then evaluated simple slopes for early life stress using the min and max values of the lower and higher intervals. In the lower interval (0.0–7.7 points), simple slopes provided evidence that for each percentage increase in nature exposure, fasting glucose levels would decrease by ~0.79 mg/dL (95% CI [−1.28, −0.29], *p* = .002) at the min value, to ~0.30 mg/dL (95% CI [−0.60, −0.01], *p* = .049) at the max value. In the higher interval (27.5–69.0 points), simple slopes provided evidence that for each percentage increase in nature exposure, fasting glucose levels would decrease by ~0.46 mg/dL (95% CI [−0.92, −0.01], *p* = .049) at the min value, to ~7.43 mg/dL (95% CI [−12.16, −2.70], *p* = .002) at the max value. In addition to this, we also sequenced simple slopes for early life stress across the observed range of the total sample (0.0–69.0 points) using units of standard deviation (Table 2). However, given the width of the confidence intervals at higher levels of early life stress, point estimates for simple slopes above two standard deviations should be interpreted with caution.

**Table 2:** Simple slopes for early life stress on the nature-glucose association.

Level	Score	Slope	SE	95% CI	P
−1 SD	0.78	−0.724	0.233	−1.183, −0.266	.002
M (x̄)	11.64	−0.170	0.167	−0.499, 0.158	.308
+1 SD	22.49	−0.230	0.212	−0.649, 0.188	.279
+2 SD	33.35	−0.903	0.312	−1.517, −0.288	.004
+3 SD	44.20	−2.189	0.672	−3.513, −0.866	.001
+4 SD	55.06	−4.088	1.285	−6.617, −1.558	.002
+5 SD	65.92	−6.601	2.124	−10.783, −2.419	.002

Simple slopes for early life stress sequenced across the observed range of the total sample in standard deviation units. Standard deviation units are relative to the sample mean (11.64 points) ± one standard deviation (10.86 points). In terms of the distribution, 74% of the sample (*n* = 254) was within one standard deviation of the mean, another 21% (*n* = 72) was within two standard deviations, and another 5% (*n* = 16) was more than two standard deviations above the mean. Estimates reflect changes in fasting glucose levels (mg/dL) relative to a 1% increase in nature exposure.

### Buffer sizes and sensitivity analyses

We then tested the two-way interaction term (Early Life Stress x Nature Exposure) across different buffer sizes for residential nature exposure and observed effects consistent with the 250 m buffer at the 500 m and 1000 m buffers. However, the significance of the moderation intervals was attenuated at larger buffer sizes (S2 Table). We then tested the sensitivity of our results using models with outliers and models unadjusted for covariates. In the models with outliers, we observed consistent effects at the 250 m, 500 m, and 1000 m buffers. However, the higher moderation interval was no longer significant at the 1000 m buffer (S2 Table). In the unadjusted models, we observed consistent effects at the 250 m, 500 m, and 1000 m buffers. However, the higher moderation interval was attenuated, and both moderation intervals were no longer significant at the 1000 m buffer (S2 Table). We then included adult stressor exposure as another covariate in the main models and observed consistent effects at the 250 m, 500 m, and 1000 m buffers (S2 Table).

## Discussion

In this cross-sectional investigation, we observed partial support for our hypothesis that adult participants with higher exposure to early-life stressors would exhibit stronger protective effects from residential nature exposure, as evidenced by lower levels of fasting blood glucose. Specifically, in a theory-guided exploration, we found that relative to the sample average, participants with higher early-life stressor exposure exhibited lower levels of fasting glucose when living in greener neighborhoods. However, we also found that relative to the sample average, participants with lower early-life stressor exposure also exhibited lower levels of fasting glucose when living in greener neighborhoods. By contrast, for participants with relatively moderate early-life stressor exposure, there was no association between neighborhood greenness and fasting glucose. Although the direction of our findings remained consistent across different buffer sizes for residential nature exposure (250 m, 500 m, and 1000 m), there was a decay of these effects with increasing buffer sizes (at a greater distance from the home address), a trend that was more evident among participants with higher early-life stressor exposure.

These findings reveal a complex interplay between social and physical environmental factors in relation to metabolic outcomes. Curiously, our hypothesis that participants with higher exposure to early-life stressors would exhibit stronger protective effects from nature exposure was supported only for participants above the sample average. This unexpected pattern raises key questions about the mechanisms underlying these associations and warrants further investigation.

### Theoretical implications

In the context of our framework, we expected that environmental sensitivity induced by early life stress could promote greater health benefits from nature exposure. This was based on mounting evidence indicating that early life stress can induce a lifelong susceptibility to stress (i.e., the Biological Embedding Model [24–33]), and that susceptibility to stress could reflect increased responsivity to both stressors and health-protective exposures (i.e., the Differential Susceptibility Hypothesis [34–41]). Therefore, we expected that the association between early life stress and environmental sensitivity would be linear, increasing the protective effects of residential nature exposure in a dose-response pattern.

However, we found that participants with higher but also lower exposure to early-life stressors both exhibited stronger protective effects from nature exposure, relative to participants near the sample average, suggesting a curvilinear U-shaped association between early life stress and environmental sensitivity. We also observed distinct patterns across our buffer sizes (at a further distance from the home address) for participants with higher versus lower exposure to early-life stressors, suggesting these may be different phenotypes of environmental sensitivity. Notably, the idea that adverse and supportive early-life environments can both induce different (ecologically adaptive) phenotypes of environmental sensitivity is well-grounded within evolutionary-developmental perspectives [67–72].

In short, these perspectives posit that: (1) early-life environments with high stressor exposure and low support upregulate a phenotype of environmental sensitivity that is adaptive in adverse environments, increasing the capacity of an individual to identify and respond to external challenges and threats; (2) early-life environments with low stressor exposure and especially high support upregulate a phenotype of environmental sensitivity that is adaptive in supportive environments, enhancing sensitivity to social resources and ambient support; (3) and by contrast, early-life environments with moderate exposures in either direction downregulate environmental sensitivity; protecting individuals against the risks of chronic stressors in environments that are not particularly adverse or consistently supportive, as experienced by the vast majority of individuals (for an excellent review on the U-shaped association, see the Biological Sensitivity to Context Theory [67–69], for the adaptive phenotypes, see the Adaptive Calibration Model [70–72]).

In the context of our framework, we expected that environmental sensitivity induced by early life stress could be a mechanism underpinning epidemiological evidence that groups in lower versus higher socioeconomic positions exhibit greater health benefits from nature exposure. However, another mutually inclusive explanation is that groups in lower socioeconomic positions with higher early-life stressor exposure are at a heightened risk of disease and mortality [133,134], and therefore have more “room to improve” from health-protective environmental exposures. This heightened risk is attributed, in part, to susceptibility to stress induced by early-life stress (e.g., a pro-inflammatory immune state [52–57]) and is further exacerbated by high exposure to stressors across the lifespan, often as a result of compromised access to the social determinants of health [59–66].

In other words, while both adverse and supportive early-life environments may induce environmental sensitivity (i.e., the Biological Sensitivity to Context Theory [67–69]), these phenotypes likely differ in their baseline physiological state and, consequently, their capacity for observable health effects (i.e., the Adaptive Calibration Model [70–72]). In turn, this suggests that a pro-inflammatory state could be a measure of environmental sensitivity specific to groups with higher early-life stressor exposure [85–88]. This interpretation aligns with recent experimental findings, demonstrating that a pro-inflammatory state is associated with stronger protective effects from a nature versus office environment (greater autonomic recovery from an acute stressor in a dose-response pattern [15]) and receiving positive versus neutral social feedback on a pre-recorded job interview (heightened neural activity in reward processing regions [78]).

Although our interpretations throughout this section are theoretical and speculative, they underscore the complexity of the moderation effect of early life stress and emphasize the need for further research to disentangle the intricate interplay between early life stress, nature exposure, and metabolic outcomes.

### Implications for research and public health

Overall, our findings contribute to growing evidence indicating that the health-protective effects of nature exposure are not the same for everyone, but can vary significantly across individuals, groups, and populations [1–7]. Specifically, we found that early life stress moderated the impact of nature exposure in a way that either promoted or diminished protective effects on fasting glucose levels. Hence, we urge future researchers to account for individual differences in life stress when investigating the protective effects of nature exposure. Failure to account for these differences could not only lead to replication challenges but also hinders our capacity to disentangle the complex role of life stress in nature and health relationships. By incorporating measures that quantify exposure to stressors across the lifespan, researchers can gain deeper insights into the mechanisms through which nature exposure influences health and well-being across diverse populations and socioeconomic gradients.

If future research corroborates these findings, this could necessitate a significant shift in how we conceptualize “susceptibility” in public health research. Traditionally, susceptibility has been primarily understood as a heightened neurobiological predisposition to the health risks of adverse environments, which could be a function of early-life exposures. However, our findings suggest a more nuanced understanding: that the same early-life exposures that increase sensitivity to adverse environments might also increase sensitivity to protective and supportive environments. Therefore, this bidirectional responsiveness might be more accurately conceptualized as a form of total “environmental sensitivity” rather than the unidirectional distinction of “susceptibility”.

This reconceptualization ultimately proposes that individuals identified in various investigations as being more reactive to adverse conditions might also be more responsive to protective and supportive conditions. In other words, susceptible individuals may have a wider range of reaction norms, covering the full gamut of environmental exposures, whereas non-susceptible individuals may have a much narrower range of reaction norms, responding less to both adverse and protective environmental exposures. Therefore, certain aspects of what is currently recognized as susceptibility from an environmental health perspective might in some instances be better framed as increased sensitivity to both adverse and protective environments. This understanding, which is grounded within the concept of differential susceptibility in developmental psychology [34–41], could have widespread and far-reaching implications for public health and environmental health research, particularly in elucidating the differential impacts of physical and social environmental factors across diverse populations and socioeconomic gradients to inform targeted interventions.

Our findings also support the value of further investigation into the idea that early life stress could be one mechanism underpinning epidemiological observations that nature exposure is associated with better health among groups in lower versus higher socioeconomic positions [1–7]. If future research corroborates these findings, this might further indicate that incorporating nature exposure into disadvantaged neighborhoods could be a strategic intervention target to curb disparities in health across socioeconomic gradients. For instance, incorporating nature exposure into residential settings is often a safe, feasible, sustainable, and cost-effective intervention target [135–138] with potential as a complementary health approach that: (1) could be installed as a passive intervention, (2) is a long-term intervention, promoting generational health, (3) could provide multiple co-benefits, and (4) could be implemented through public health policy [138].

### Strengths and limitations

To our knowledge, this is the first investigation to provide evidence suggesting that early life stress is associated with greater health benefits from nature exposure. While modest, these findings are supported by several study strengths, including our use of a comprehensive measure to quantify early life stress [96], the integration of various well-established theories across diverse fields to support our claims [52,55,67,70,73,79,81,83], and our focus on biomarkers of glucose dysregulation, a risk factor for type 2 diabetes that significantly contributes to the global burden of disease [97,98]. Identifying a significant moderation effect of early life stress on the nature-glucose association at particularly low levels of nature exposure also increases our confidence in a robust association across these social and physical environmental factors.

At the same time, several limitations should be considered. The use of a cross-sectional, observational design limited causal inference and our associated claims, while theoretically grounded, remain speculative until corroborated in future studies. As our sample primarily consisted of White Hispanic females who lived in West Texas, the representativeness of our study was limited in terms of race and ethnicity, biological sex and gender, and regional context. Because these social identity factors can shape stressor exposure, access to nature, and biological responses to these exposures [59,60], additional research is needed to investigate the generalizability of the present findings to other populations. Future investigators are also encouraged to examine the influence of family dynamics on these associations, as the literature supporting our framework suggests it is not just early life stress, but the ratio of early life stress versus support that ultimately signals the development of different adaptive phenotypes of environmental sensitivity [67,70]. Notably, the quality and perceived impact of maternal relationships may be a particularly salient factor, as highly supportive relationships have been shown to protect against the development of susceptibility to stress during adulthood [139].

Although the Normalized Difference Vegetation Index is a reliable measure of nature exposure [123], this measure does not distinguish between different types or account for the quality, physical accessibility, safety, or perceived usability of the exposure, which could be important moderating factors. In addition, subjective experiences with nature could also moderate biological responses and may be particularly relevant in regions with limited access to natural greenspace [140]. The interpretation of our findings was based on long-term exposure to residential nature; however, without complete information on residential histories or time spent away from home, we were constrained to cumulative exposure estimates using the current residential address of each participant. Yet, considering that participants lived at their reported address for more than a decade, on average, and that spatial variation of green nature is limited in desert regions, these limitations are likely non-differential with respect to our findings.

There was also a large amount of missing data for the early life stress assessment (22%), resulting from time constraints during the laboratory visit due to the length of the comprehensive assessment (≥ 20 min). Therefore, these data are considered to be missing completely at random, and no significant differences were observed between participants missing data and the rest of the sample across our study variables (S3 Table). While the Stress and Adversity Inventory for Adults is well-validated [96,119–121], we were unable to provide sample-specific reliability estimates without item-level data. We also did not collect data on acute exposures before the laboratory visit which may have influenced fasting glucose assessments. Although we adjusted for a range of covariates to eliminate the most plausible alternative explanations and confounders (e.g., physical activity, weight loss), it is possible that other unassessed factors might have contributed to our findings (e.g., maternal relationships and attachment). Looking forward, future studies with more representative samples, more rigorous assessment of nature exposure and other potential confounders, and longitudinal designs to support causal modeling will be helpful.

### Conclusion

Ultimately, future research in line with this theoretical framework could lead to a greater understanding of the ways in which nature-based interventions could be leveraged to attenuate health disparities among vulnerable populations. Specifically, this could further underscore the value of integrating protective physical environments into public health strategies, especially for groups with higher exposure to early-life stressors, who are particularly susceptible to health risks but also might stand to experience the greatest health benefits from nature exposure. As the evidence for this framework expands, we could more effectively identify and target mechanisms of health risks and benefits among susceptible groups and tailor public health interventions, taking into account individual differences in environmental sensitivity to attenuate health disparities across socioeconomic gradients.

## Supporting information

S1 Fig Bivariate correlation plot. Bivariate correlations across our included measures. Diagonal crosses are superimposed over non-significant correlations. Correlations for nature exposure were consistent across radial buffer sizes (250 m, 500 m, and 1000 m).
(TIF)

S1 Table Overview of epidemiological evidence for nature exposure and health disparities. Health disparity gaps refer to the difference in health outcomes between lower and higher socioeconomic groups. Socioeconomic groups were determined using area-level income deprivation, census-level neighborhood-median household income, and self-reported income, financial strain, and educational attainment. Residential nature exposure was quantified using satellite and street-view imagery, land-cover databases, and self-reported access. Health outcomes were assessed using national databases, clinical records, biomarkers, and self-reported measures.
(DOCX)

S2 Table Estimates across all models. Estimates were obtained from continuous two-way interaction terms used to assess the moderation effect of early life stress on the association between residential nature exposure and fasting glucose levels across different buffer sizes. Interaction terms were probed using Johnson-Neyman (J-N) intervals to determine the specific regions along the continuum of early life stress that significantly moderate the nature-glucose association. Interaction terms were tested across models: (1) adjusted for covariates and excluding outliers, (2) adjusted for covariates and including outliers, (3) unadjusted for covariates and excluding outliers, and (4) adjusted for covariates, excluding outliers, and adjusted for adult stressor exposure. Outliers included nine participants identified through studentized residuals. Simple slopes are shown at the min and max values (points) of the significant region for the lower and higher intervals and reflect changes in fasting glucose levels (mg/dL) relative to a 1% increase in nature exposure. The vertex refers to the turning point (the score at the top of the curve) where the moderation effect changed direction. ** *p* < .01, * *p* < .05.
(DOCX)

S3 Table Participant demographics by STRAIN status (missing vs completed). ^1^ After excluding participants older than 40 years of age (*n* = 9), missing nature exposure data (*n* = 4), and missing fasting glucose data (*n* = 18), 114 participants were excluded for missing STRAIN data. ^2^ After excluding participants older than 40 years of age (*n* = 10), missing nature exposure data (*n* = 12), and missing fasting glucose data (*n* = 10), 340 participants were included in the final analytical sample. ^3^ One-way test assuming unequal variance or chi-square test.
(DOCX)
