# Supplementary figures and images for: Early life stress enhances the association between residential nature exposure and fasting blood glucose
Source: PLoS One. 2026 Jul 9;21(7):e0352771. doi: 10.1371/journal.pone.0352771 (PMC13349149; doi:10.1371/journal.pone.0352771)

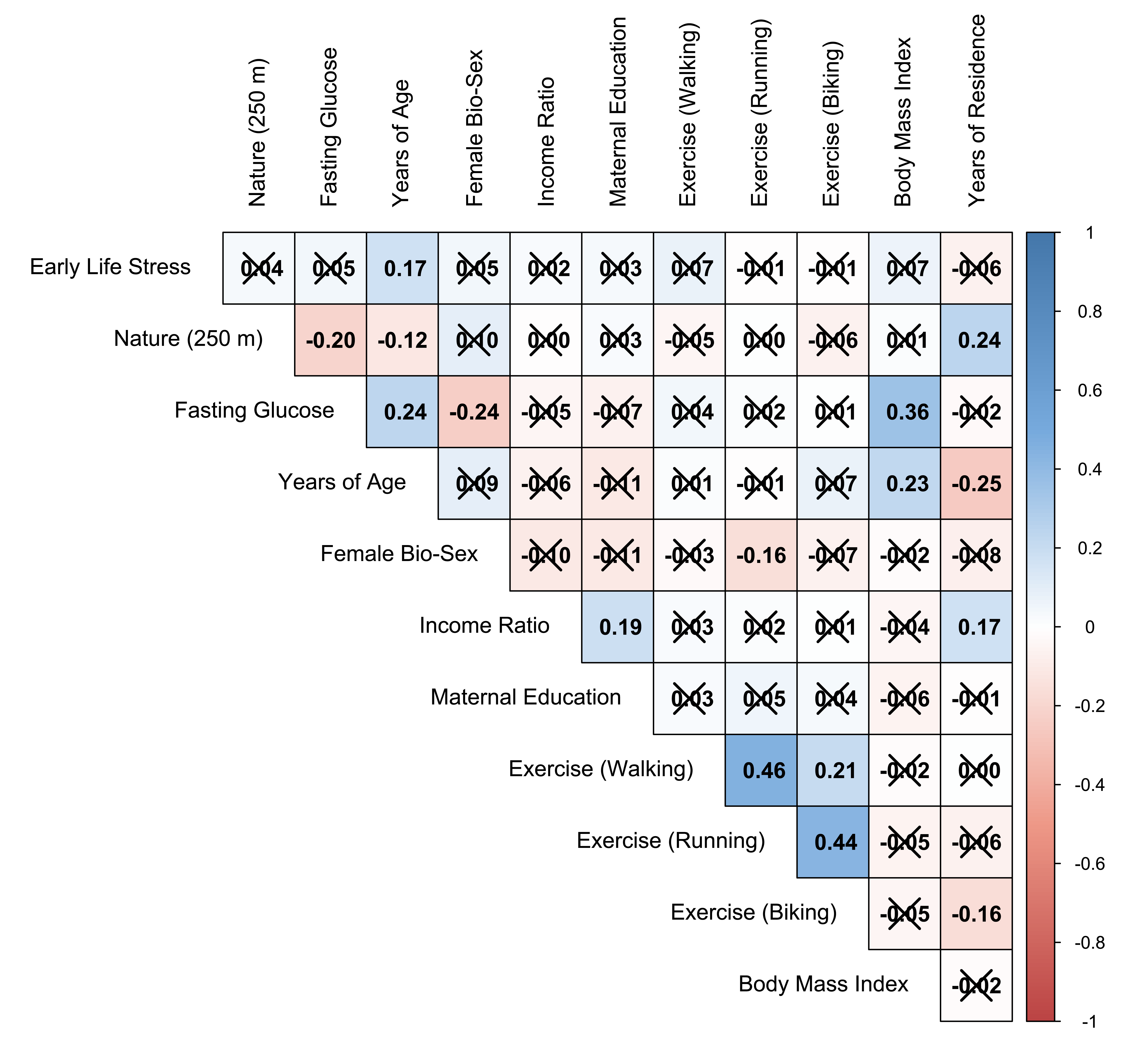

Supplement: S1 Fig — Bivariate correlations across our included measures. Diagonal crosses are superimposed over non-significant correlations. Correlations for nature exposure were consistent across radial buffer sizes (250 m, 500 m, and 1000 m). (TIF) [file pone.0352771.s001.tif]
