# Supplementary material for: Early life stress enhances the association between residential nature exposure and fasting blood glucose
Source: PLoS One. 2026 Jul 9;21(7):e0352771. doi: 10.1371/journal.pone.0352771 (PMC13349149; doi:10.1371/journal.pone.0352771)
Supplement: S1 Table — Health disparity gaps refer to the difference in health outcomes between lower and higher socioeconomic groups. Socioeconomic groups were determined using area-level income deprivation, census-level neighborhood-median household income, and self-reported income, financial strain, and educational attainment. Residential nature exposure was quantified using satellite and street-view imagery, land-cover databases, and self-reported access. Health outcomes were assessed using national databases, clinical records, biomarkers, and self-reported measures. (DOCX) [file pone.0352771.s002.docx]

**S1 Table. Overview of Epidemiological Evidence for Nature Exposure and Health Disparities**

| **Study** | **Location** | **Population** | **Health Outcome(s)** | **Findings** |
| --- | --- | --- | --- | --- |
| Mitchel &  Popham,  2008 | England | Population of England below retirement age (*n* = 40,813,236) | All-cause mortality and deaths from circulatory disease, lung cancer, and intentional self-harm | Smaller health disparity gaps for all-cause mortality and deaths from circulatory disease in greener neighborhoods |
| Mitchel et al., 2015 | 34 European nations | Participants in the European Quality of Life Survey (*n* = 21,294) | Mental well-being | Smaller health disparity gaps for mental well-being in greener neighborhoods |
| Brown et al.,  2016 | Miami-Dade County, Florida | Medicare beneficiaries (*n* = 249,405) | Chronic conditions, diabetes, hypertension, and hyperlipidemia | Smaller health disparity gap for chronic conditions in greener neighborhoods |
| Brown et al.,  2018 | Miami-Dade County, Florida | Medicare beneficiaries (*n* = 249,405) | Alzheimer’s disease and depression | Smaller health disparity gap for depression in greener neighborhoods |
| Nicholls et al., 2022 | Scotland | Population of Scotland under 65 years of age (small-area level data) | Years of life lost | Smaller health disparity gap for years of life lost in greener neighborhoods |
| Wang et al.,  2024 | China | Participants in the 33 Chinese Community Health Study (*n* = 2,154) | Kidney failure | Smaller health disparity gap for kidney failure in greener neighborhoods |
