# Supplementary material for: Early life stress enhances the association between residential nature exposure and fasting blood glucose
Source: PLoS One. 2026 Jul 9;21(7):e0352771. doi: 10.1371/journal.pone.0352771 (PMC13349149; doi:10.1371/journal.pone.0352771)
Supplement: S2 Table — Estimates were obtained from continuous two-way interaction terms used to assess the moderation effect of early life stress on the association between residential nature exposure and fasting glucose levels across different buffer sizes. Interaction terms were probed using Johnson-Neyman (J-N) intervals to determine the specific regions along the continuum of early life stress that significantly moderate the nature-glucose association. Interaction terms were tested across models: (1) adjusted for covariates and excluding outliers, (2) adjusted for covariates and including outliers, (3) unadjusted for covariates and excluding outliers, and (4) adjusted for covariates, excluding outliers, and adjusted for adult stressor exposure. Outliers included nine participants identified through studentized residuals. Simple slopes are shown at the min and max values (points) of the significant region for the lower and higher intervals and reflect changes in fasting glucose levels (mg/dL) relative to a 1% increase in nature exposure. The vertex refers to the turning point (the score at the top of the curve) where the moderation effect changed direction. ** p < .01, * p < .05. (DOCX) [file pone.0352771.s003.docx]

|  | **Buffer Size** | **Interaction Term** | **Lower Interval (*p* < .05)** | | **Vertex** | **Higher Interval (*p* < .05)** | |
| --- | --- | --- | --- | --- | --- | --- | --- |
| 1. **Main Models** | 250 Meters | -0.0026**  (-0.0044, -0.0008) | 0.00 points  (-0.79 mg/dL) | 7.68 points  (-0.30 mg/dL) | 16.01 points | 27.46 points  (-0.46 mg/dL) | 69.00 points  (-7.43 mg/dL) |
|  | 500 Meters | -0.0030**  (-0.0051, -0.0009) | 0.00 points  (-0.81 mg/dL) | 5.73 points  (-0.35 mg/dL) | 16.37 points | 30.34 points  (-0.59 mg/dL) | 69.00 points  (-8.25 mg/dL) |
|  | 1000 Meters | -0.0024*  (-0.0044, -0.0003) | 0.00 points  (-0.64 mg/dL) | 0.97 points  (-0.57 mg/dL) | 15.97 points | 33.59 points  (-0.77 mg/dL) | 69.00 points  (-6.66 mg/dL) |
| 1. **With Outliers** | 250 Meters | -0.0025*  (-0.0048, -0.0002) | 0.00 points  (-0.94 mg/dL) | 8.44 points  (-0.39 mg/dL) | 17.26 points | 31.87 points  (-0.73 mg/dL) | 69.00 points  (-6.89 mg/dL) |
|  | 500 Meters | -0.0028*  (-0.0055, -0.0001) | 0.00 points  (-0.93 mg/dL) | 6.27 points  (-0.44 mg/dL) | 17.34 points | 37.60 points  (-1.24 mg/dL) | 69.00 points  (-7.52 mg/dL) |
|  | 1000 Meters | -0.0025  (-0.0052, 0.0002) | 0.00 points  (-1.01 mg/dL) | 6.51 points  (-0.47 mg/dL) | 19.71 points | --- | --- |
| 1. **Without Covariates** | 250 Meters | -0.0016  (-0.0035, 0.0004) | 0.00 points  (-0.65 mg/dL) | 10.45 points  (-0.34 mg/dL) | 14.68 points | 25.06 points  (-0.48 mg/dL) | 54.17 points  (-2.73 mg/dL) |
|  | 500 Meters | -0.0019  (-0.0042, 0.0004) | 0.00 points  (-0.72 mg/dL) | 7.87 points  (-0.35 mg/dL) | 16.18 points | 33.78 points  (-0.82 mg/dL) | 43.98 points  (-1.71 mg/dL) |
|  | 1000 Meters | -0.0015  (-0.0038, 0.0009) | --- | --- | 15.84 points | --- | --- |
| 1. **With Adult Stressors** | 250 Meters | -0.0026**  (-0.0044, -0.0008) | 0.00 points  (-0.77 mg/dL) | 7.23 points  (-0.31 mg/dL) | 15.94 points | 27.64 points  (-0.47 mg/dL) | 69.00 points  (-7.51 mg/dL) |
|  | 500 Meters | -0.0031**  (-0.0051, -0.0010) | 0.00 points  (-0.79 mg/dL) | 5.32 points  (-0.35 mg/dL) | 16.22 points | 30.16 points  (-0.59 mg/dL) | 69.00 points  (-8.50 mg/dL) |
|  | 1000 Meters | -0.0025*  (-0.0046, -0.0004) | 0.00 points  (-0.64 mg/dL) | 0.79 points  (-0.58 mg/dL) | 15.85 points | 33.00 points  (-0.75 mg/dL) | 69.00 points  (-7.05 mg/dL) |

**S2 Table. Estimates Across All Models**
