# Supplementary material for: Early life stress enhances the association between residential nature exposure and fasting blood glucose
Source: PLoS One. 2026 Jul 9;21(7):e0352771. doi: 10.1371/journal.pone.0352771 (PMC13349149; doi:10.1371/journal.pone.0352771)
Supplement: S3 Table — 1 After excluding participants older than 40 years of age (n = 9), missing nature exposure data (n = 4), and missing fasting glucose data (n = 18), 114 participants were excluded for missing STRAIN data. 2 After excluding participants older than 40 years of age (n = 10), missing nature exposure data (n = 12), and missing fasting glucose data (n = 10), 340 participants were included in the final analytical sample. 3 One-way test assuming unequal variance or chi-square test. (DOCX) [file pone.0352771.s004.docx]

**S3 Table. Participant Demographics by STRAIN Status (Missing vs Completed)**

| **Characteristic** | **M ± (SD) or N (%)** | | | **p-value ^3^** |
| --- | --- | --- | --- | --- |
|  | **Total Sample**  **(*n* = 517)** | **Missing STRAIN ^1^**  **(*n* = 145)** | **Completed STRAIN ^2^**  **(*n* = 372)** |  |
| **Demographics** |  |  |  |  |
| Years of Age | 25.00 ± 6.12 | 25.88 ± 7.11 | 24.65 ± 5.67 | .065 |
| Female Bio-Sex | 413 (80%) | 124 (86%) | 289 (78%) | .061 |
| Income Ratio | 2.32 ± 1.87 | 2.37 ± 1.83 | 2.30 ± 1.89 | .725 |
| Maternal Education | 3.57 ± 1.30 | 3.46 ± 1.37 | 3.60 ± 1.28 | .302 |
| **Physical Activity** |  |  |  |  |
| Walking | 4.18 ± 1.58 | 4.08 ± 1.57 | 4.22 ± 1.59 | .377 |
| Running | 2.62 ± 1.96 | 2.47 ± 1.87 | 2.67 ± 1.99 | .285 |
| Biking | 0.98 ± 1.71 | 1.04 ± 1.59 | 0.95 ± 1.75 | .612 |
| **Health Indicators** |  |  |  |  |
| Body Mass Index | 25.51 ± 5.37 | 25.53 ± 5.19 | 25.50 ± 5.43 | .956 |
| Glucose (mg/dL) | 90.97 ± 10.93 | 90.29 ± 10.89 | 91.21 ± 10.95 | .417 |
| **Nature Exposure** |  |  |  |  |
| NDVI (250 m) | 10.55 ± 3.11 | 10.22 ± 2.94 | 10.68 ± 3.17 | .123 |
| NDVI (500 m) | 10.68 ± 2.87 | 10.48 ± 2.95 | 10.75 ± 2.84 | .358 |
| NDVI (1000 m) | 10.77 ± 2.61 | 10.65 ± 2.73 | 10.82 ± 2.56 | .506 |
| Years of Residence | 10.73 ± 8.42 | 11.57 ± 9.37 | 10.40 ± 8.02 | .187 |
